# Tumor infiltrating immune cells (TIICs) as a biomarker for prognosis benefits in patients with osteosarcoma

**DOI:** 10.1186/s12885-020-07536-3

**Published:** 2020-10-21

**Authors:** Ying Chen, Bo Zhao, Xiaohu Wang

**Affiliations:** 1 Department of Ultrasound, Xiaoshan Traditional Chinese Medical Hospital, Hangzhou, 311200 China; 2 Department of Orthopaedic, Hanchuan People’s Hospital, Hanchuan, 311200 Hubei Province China

**Keywords:** Osteosarcoma, TIICs, Prognosis, Immune risk score model, Nomogram

## Abstract

**Background:**

Osteosarcoma is a rare malignant bone tumor in adolescents and children. Poor prognosis has always been a difficult problem for patients with osteosarcoma. Recent studies have shown that tumor infiltrating immune cells (TIICs) are associated with the clinical outcome of osteosarcoma patients. The aim of our research was to construct a risk score model based on TIICs to predict the prognosis of patients with osteosarcoma.

**Methods:**

CIBERSORTX algorithm was used to calculate the proportion of 22 TIIC types in osteosarcoma samples. Kaplan-Meier curves were drawn to investigate the prognostic value of 22 TIIC types. Forward stepwise approach was used to screen a minimal set of immune cell types. Multivariate Cox PHR analysis was performed to construct an immune risk score model.

**Results:**

Osteosarcoma samples with CIBERSORTX output *p* value less than 0.05 were selected for research. Kaplan-Meier curves indicated that naive B cells (*p* = 0.047) and Monocytes (*p* = 0.03) in osteosarcoma are associated with poor prognosis. An immune risk score model was constructed base on eight immune cell types, and the ROC curve showed that the immune risk score model is reliable in predicting the prognosis of patients with osteosarcoma (AUC = 0.724). Besides, a nomogram model base on eight immune cell types was constructed to predict the survival rate of patients with osteosarcoma.

**Conclusions:**

TIICs are closely related to the prognosis of osteosarcoma. The immune risk score model based on TIICs is reliable in predicting the prognosis of osteosarcoma.

## Background

Osteosarcoma is a primary malignant bone tumor commonly seen in adolescents and children, with an incidence of 8–11 per million in the 15–19 age group [1–3]. Osteosarcoma is characterized by early lung metastasis and poor prognosis, which seriously threaten the physical and mental health of patients. Currently, chemotherapy along with surgery is the main clinical treatment, which has improved the prognosis of osteosarcoma. However, recurrence or metastasis remain the poor prognosis [4–7]. Therefore, it is crucial to search for meaningful prognostic biomarker to improve the survival rate of patients.

Recent advances in the study of tumor microenvironment (TME) have opened up new directions for the study of osteosarcoma [8, 9]. TME is mainly composed of tumor cells, intercellular matrix, blood vessels and TIICs [10–12]. Recent studies have found a significant correlation between the TIICs in TME and prognosis in cancer patients. For example, in colorectal cancer, Angell H et al. [13] graded the density of CD3 + T and CD8 + T cells at the center of the tumor and tumor infiltration border area, and added the scores to obtain the total immune score (0 to 4 points). Patients were divided into 5 stages according to the total immune score. Further clinical data analysis confirmed that the immune score model had a better prognostic differentiation effect than the TNM staging system. However, the construction of the model did not fully integrate with the TIICs in the tumor. Research by Zhou R et al. [14] makes up for this limitation by using the CIBERSORTX algorithm to estimate the proportion of 22 TIIC types in samples from six microarray datasets. An immune risk score model was constructed based on 22 TIIC types, which showed better prognostic value than the TNM staging system. Although a large number of studies have explored the prognostic value of TIICs in osteosarcoma [15–17], there has been no study to construct a prognostic risk score model based on immune cells. In our research, we also use CIBERSORTX algorithm to calculate the proportion of 22 TIIC types in samples from three microarray datasets containing prognostic information of osteosarcoma. An immune risk score model was constructed based on TIIC types selected by forward stepwise approach to provide more valuable biomarkers for the prognosis of osteosarcoma.

## Methods

### Data acquisition

To obtain gene expression microarray of osteosarcoma and corresponding clinical data, we used “osteosarcoma” and “prognosis” in the search box of the gene expression omnibus (GEO, https://www.ncbi.nlm.nih.gov/geo/) database as keywords for joint retrieval [18]. After filtering, data sets GSE16091 [19], GSE21257 [20] and GSE39055 [21] were selected as training dataset for this research. Clinical data with prognostic information were extracted directly from the matrix file on the page of the corresponding gene chip in the GEO database. Then we preprocessed the gene expression profile data from different detection platforms and converted the gene probe name into the official gene name. The testing dataset Target-OS was obtained from the Target database (https://ocg.cancer.gov/programs/target).

### Evaluation of TIICs

After processing the chip, we open the CIBERSORTX website (http://CIBERSORTx.stanford.edu/), upload the processed chip data, and click to run [22–25]. At the end of the run, CIBERSORTX prints out a table containing the proportion of 22 immune cell types in each sample. CIBERSORTX will analyze each sample and calculate a *p* value through Monte Carlo algorithm, which represents the credibility of the results. When p is less than 0.05, it indicates that the proportion of 22 TIIC types analyzed by CIBERSORTX is accurate. Therefore, in this study, only samples with CIBERSORTX output p value less than 0.05 were selected for subsequent analysis as Christine Desmedt et al. [26] described previously. It should be noted that the output of CIBERSORTX is relative composition ratio. Therefore, the sum of the composition ratio of 22 immune cell types in each sample is 1.

### Statistical analyses

All statistical analyses in this study were carried out in R 3.5.3 and SPSS 23.0. All the analyses were performed by bilateral test, and a *p* value less than 0.05 was considered statistically significant. One-way ANOVA was used for comparison between groups of continuous variables [27]. Pearson’s correlation analysis and spearman’s correlation analysis was used in the correlation analysis [28]. Kaplan-Meier curves were drawn to show the survival of patients, and Log-rank method was used to test whether there were differences [29]. The time-dependent receiver operating characteristic (ROC) curve was used to analyze the sensitivity and specificity of the prognostic model [30]. Multivariate analysis was used to analyze the independence of immune cells as prognostic factors. Nomogram model was constructed to predict the recurrence rate of patients with osteosarcoma [31].

### Establishment and confirmation of an immune risk score model

Firstly, forward stepwise approach was used to screen a more effective immune cell set at 22 TIIC types to construct a prognostic model [32]. Then, all of the selected immune cell types were fitted into multivariate Cox PHR model to establish an immune risk score model. Each sample will receive a risk score according to the immune risk score model. The samples were divided into high-risk and low-risk groups according to the cutoff value of the risk score. The Kaplan-Meier curve was drawn to compare the recurrent risk between high-risk and low-risk groups. The time-dependent receiver operating characteristic (ROC) curve to identify the reliability of the immune risk score model in predicting recurrent risk [33]. Then, we verified the model for Target-OS dataset. Patients in Target-OS dataset were divided into high- risk and low-risk groups based on the same cutoff value as GEO dataset to test the prediction ability of the immune risk score model [34].

## Results

### The landscape of TIICs in osteosarcoma

**Fig. 1:**
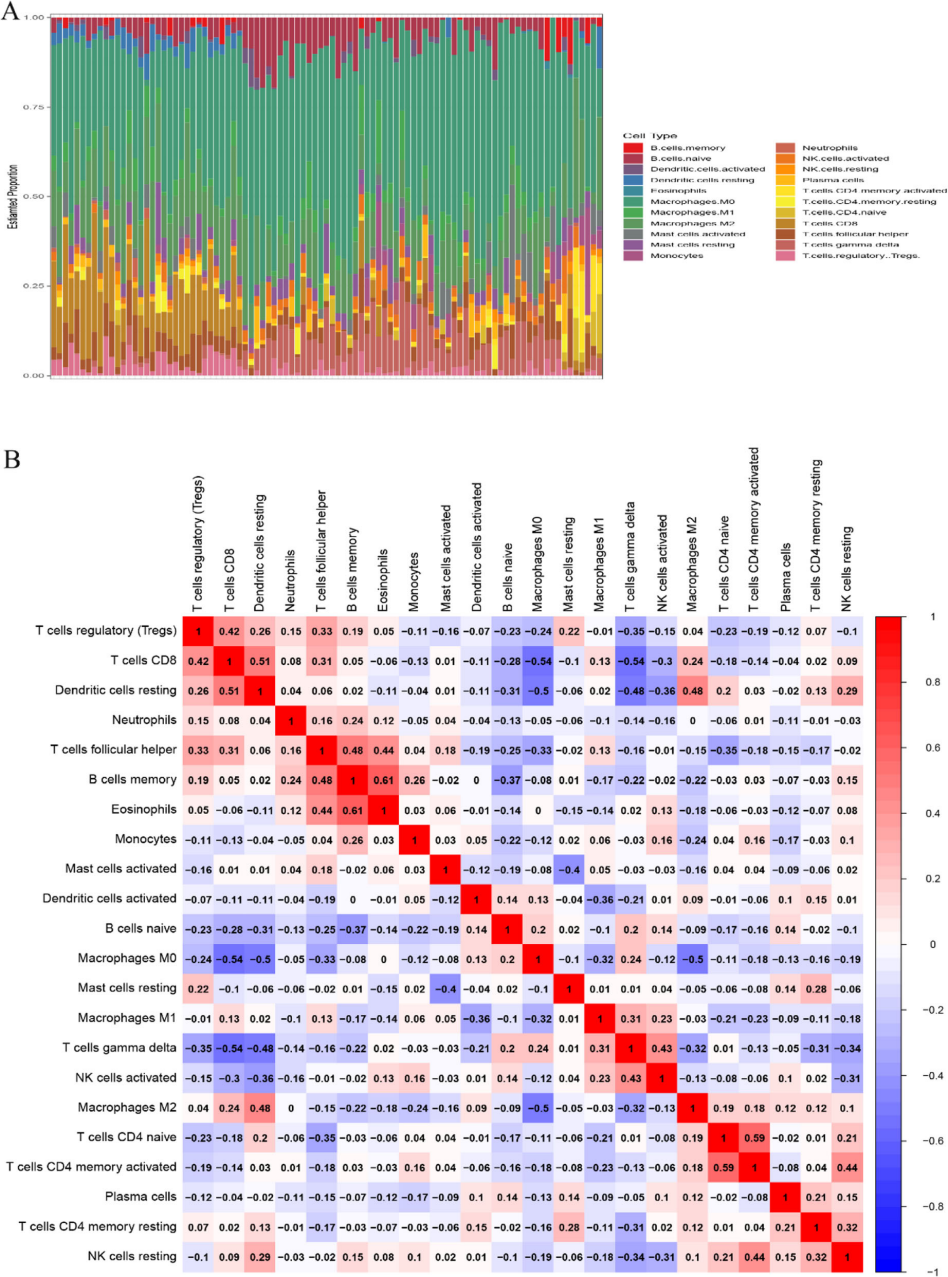
The landscape of tumor-infiltrating immune cells in osteosarcoma. **a**. The proportion of 22 immune cell types in osteosarcoma tissues. **b**. Correlation matrix between 22 immune cell types in osteosarcoma

CIBERSORTX algorithm was used to screen out samples with CIBERSORTX output *P* value less than 0.05 in GSE16091, GSE21257 and GSE39055 data sets for research. Ultimately, 95 patients with osteosarcoma was screened out (Table 1). Percentage bar chart was drawn to show the proportion of 22 TIIC types in each sample (Fig. 1a). The results revealed that the proportion of TIIC types in osteosarcoma were B cells naïve (5.14%), B cells memory (0.88%), Plasma cells (1.77%), T cellsCD8(6.43%), T cells CD4 naive (0.90%), T cells CD4 memory resting (0.74%), T cells CD4 memory activated (1.02%), T cells follicular helper (4.79%), Tregs (2.40%), T cells gamma delta (6.02%), NK cells resting (0.38%), NK cells activated (2.26%), Monocytes (1.53%), Macrophages M0 (40.54%), Macrophages M1 (3.66%), Macrophages M2(13.34%), Dendritic cells resting (1.40%), Dendritic cells activated (0.79%), Mast cells resting (2.88%), Mast cells activated (2.43%), Eosinophils (0.05%), Neutrophils (0.62%), respectively. Figure 1b indicated the correlation coefficient between 22 TIIC types. Red and blue colors indicate positive and negative correlation, respectively. Color intensity corresponds to the degree of correlation. We can find that eosinophils and memory B cells have the strongest positive correlation (*r* = 0.61), CD8 + T cells had the strongest negative correlation with M0 Macrophages and γδT cells (*r* = − 0.54).

**Table 1:** The proportional distribution of samples with different *P*-value in three datasets

	*P* < 0.05	*P* > 0.05	Total
GSE16091	33	1	34
GSE21257	52	1	53
GSE39055	10	27	37
Total	95	29	124

### Predictive value of TIICs in osteosarcoma

**Fig. 2:**
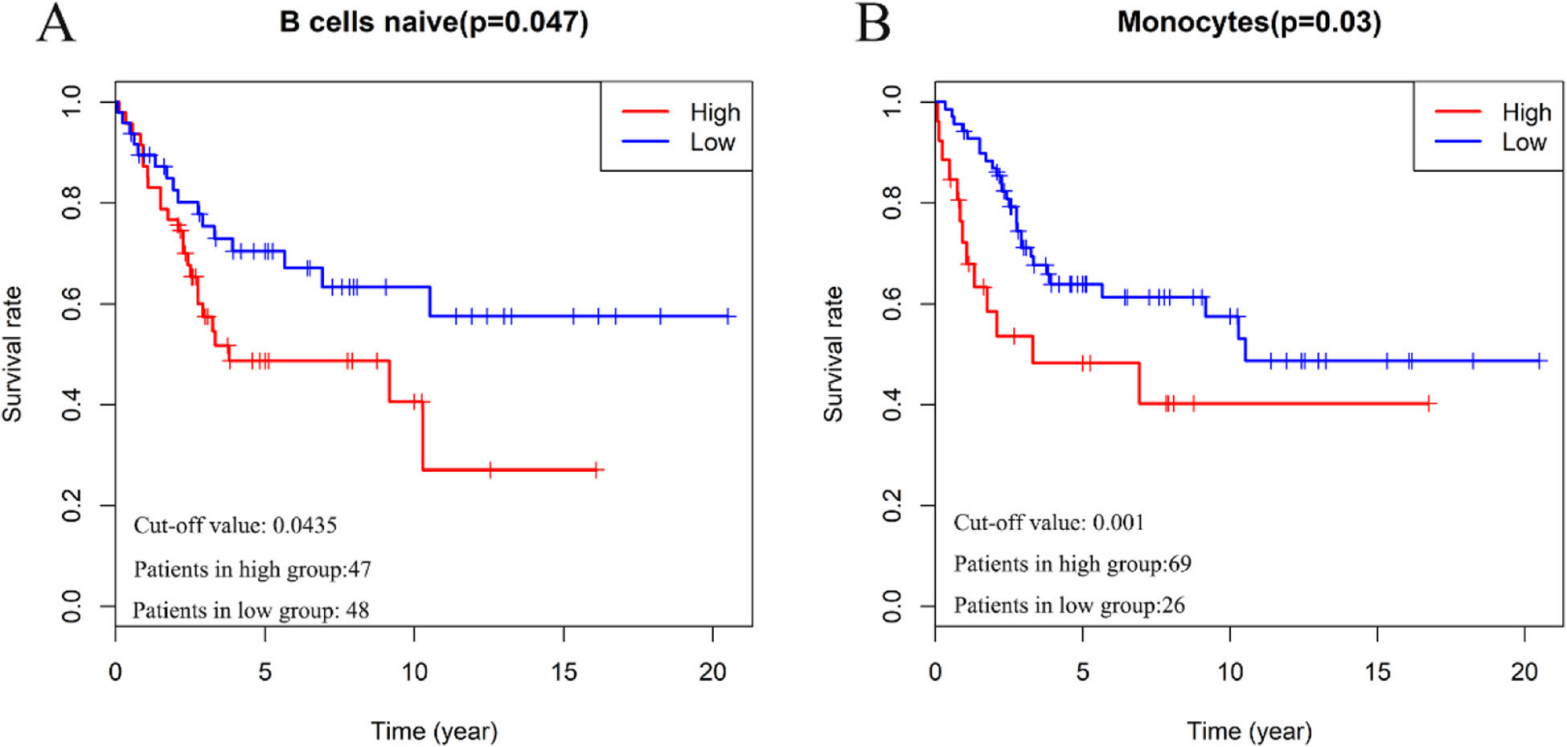
The prognostic value of 22 immune cell types in osteosarcoma by plotting Kaplan-Meier curves

To investigate the prognostic value of 22 TIIC types, Kaplan-Meier curves were drawn and the results were evaluated by log-rank test. In Fig. 2, we can find that high abundance of naive B cells (*p* = 0.047, Fig. 2a) and Monocytes (*p* = 0.03, Fig. 2b) in osteosarcoma is associated with poor prognosis.

### Establishment and confirmation of an immune risk score model

**Fig. 3:**
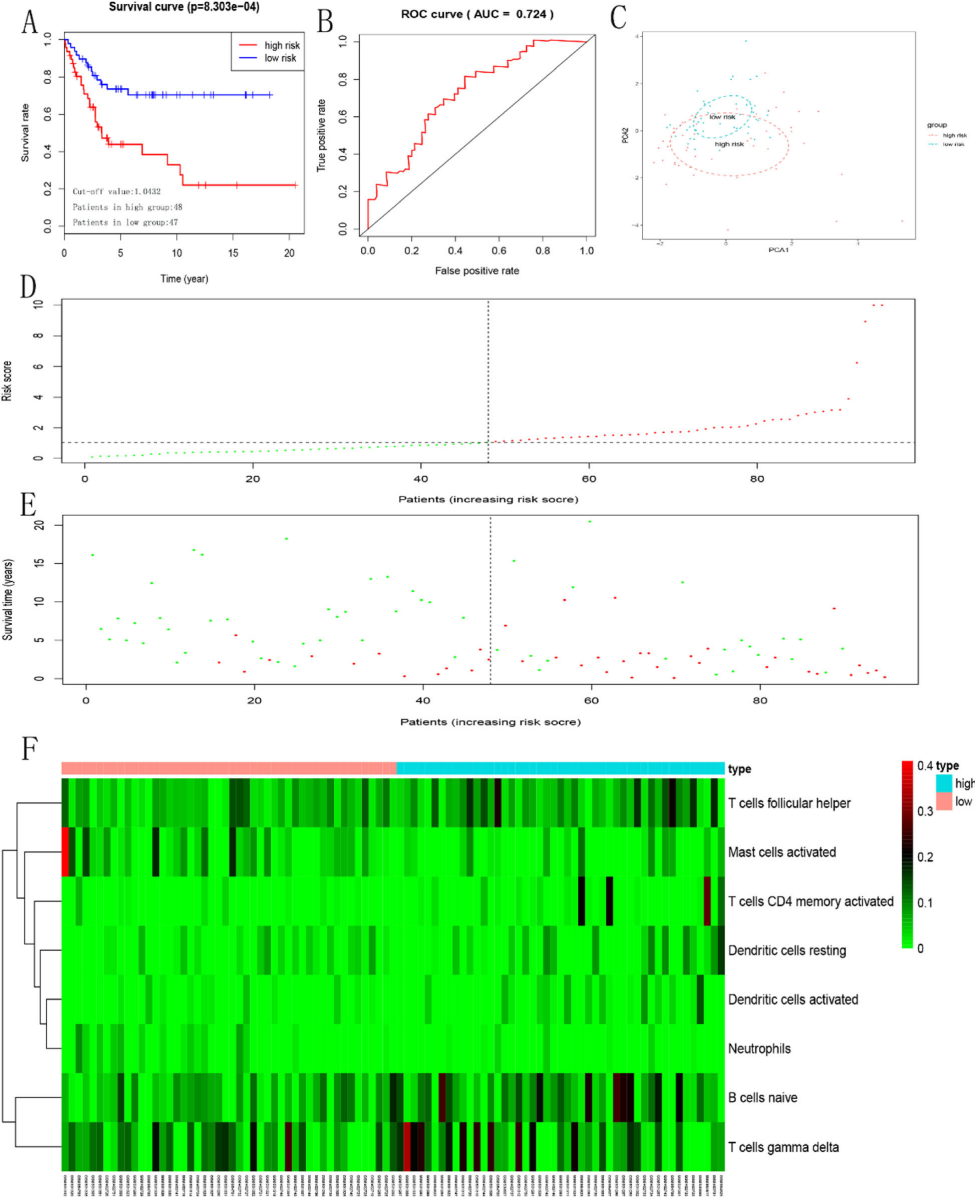
Establishment of immune risk score model. **a**. Kaplan–Meier survival curves of overall survival between high-risk and low-risk patients. **b** ROC curve AUC statistics assess the predictive power of the immune risk score model. **c** Principal component analysis distinguishes high - and low-risk groups. The distribution of patients’ risk score (**d**) and survival state (**e**), and 8 immune cell type infiltration of patients with osteosarcoma (**f**)

**Fig. 4:**
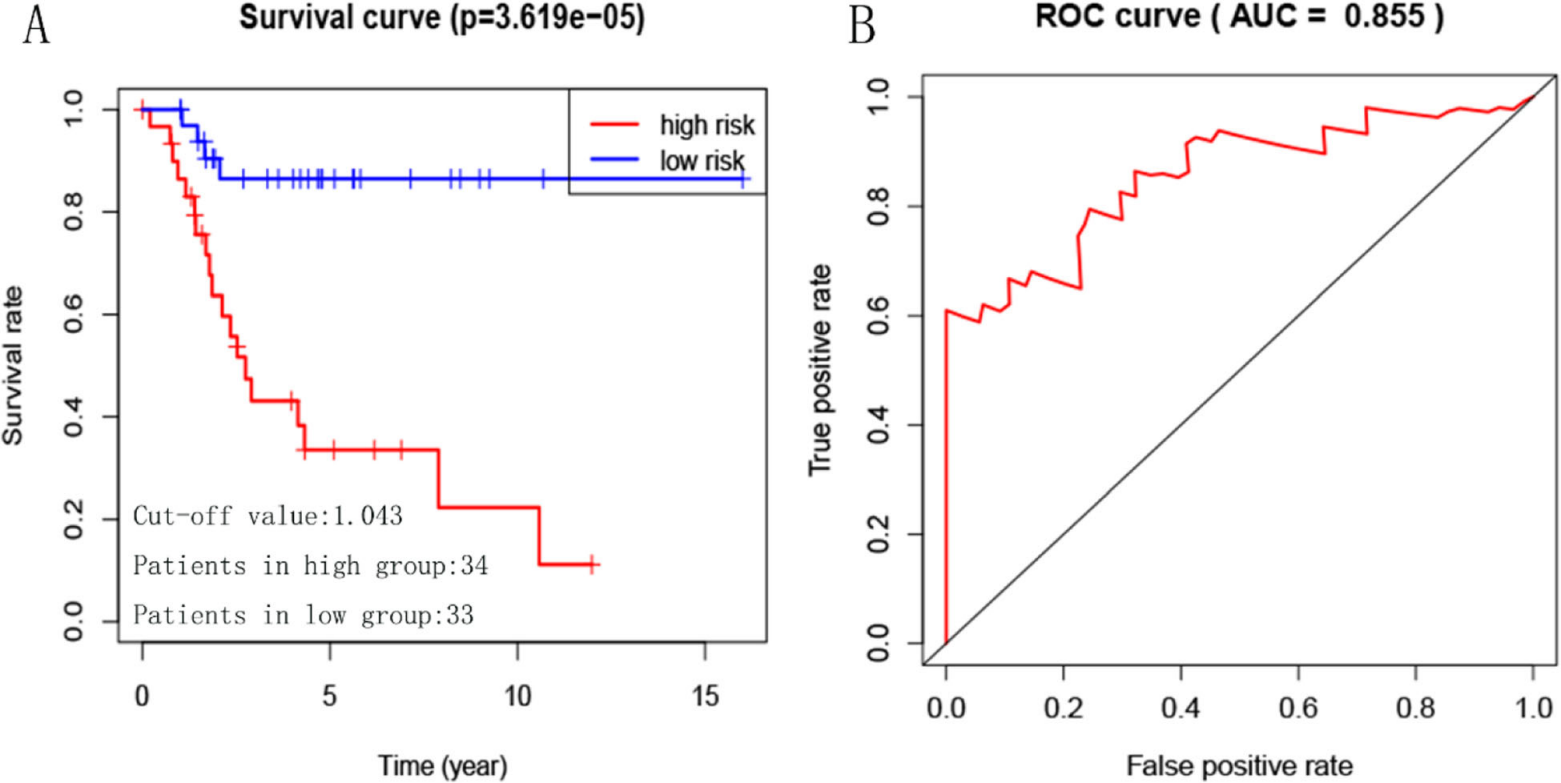
Confirmation of immune risk score model. **a** Kaplan–Meier survival curves of overall survival between high-risk and low-risk patients. **b** ROC curve AUC statistics assess the predictive power of the immune risk score model

Considering that the effect of TIICs on the prognosis of patients is not independent. We constructed an immune risk score model to evaluate the prognosis of patients individually. Firstly, we used the forward stepwise regression method to screen out a minimal set of TIIC types. Finally, eight immune cell types were selected. Then, eight immune cell types were filled into multivariate Cox PHR analysis to construct an immune risk score model. Formula is as follows: Risk_8_ = 8.49 _*_ naive B cells + 15.7 _*_ activated memory CD4 + T cells + 17.1 _*_ follicular helper T cells + 6.07 _*_ γδ T cells + 27.9 _*_ resting Dendritic cells + 24.7 _*_ activated Dendritic cells - 26.8 _*_ Neutrophils − 7.38 _*_ activated Mast cells (Table 2). Each sample will be given a risk score based on the model. Patients were divided into high- and low-risk group according to the median risk score. Kaplan-Meier curves indicated that patients in high-risk group had a poorer prognosis than those in low-risk group (*p* = 8.303e-04, Fig. 3a). The ROC curve showed that the immune risk score model is reliable in predicting the prognosis of patients with osteosarcoma (AUC = 0.724, Fig. 3b). Principal component analysis revealed that the immune risk score model can distinguish the patients into high - and low-risk groups well (Fig. 3c). In addition, Fig. 3d, e and f respectively showed the risk score, survival status and 8 immune cell types infiltration of patients with osteosarcoma. Then, the prediction ability of the immune risk score model was verified in Target-OS dataset based on the same cutoff value as GEO dataset. The result indicated that the prediction ability of the immune risk score model is reliable (Fig. 4).

**Table 2:** Multivariate Cox PHR analysis of eight immune cells. We found that naive B cells, activated memory CD4 + T cells, follicular helper T cells, resting Dendritic cells, activated Dendritic cells were independent predictors for osteosarcoma patients (*P* < 0.05)

	coef	exp (coef)	se (coef)	z	p
B.cells.naive	8.49E+ 00	4.87E+ 03	3.47E+ 00	2.451	0.014255
T.cells.CD4.memory.activated	1.57E+ 01	6.55E+ 06	5.18E+ 00	3.031	0.002435
T.cells.follicular.helper	1.71E+ 01	2.61E+ 07	5.12E+ 00	3.334	0.000855
T.cells.gamma.delta	6.07E+ 00	4.32E+ 02	3.37E+ 00	1.799	0.071947
Dendritic.cells.resting	2.79E+ 01	1.29E+ 12	8.71E+ 00	3.203	0.001359
Dendritic.cells.activated	2.47E+ 01	5.33E+ 10	9.45E+ 00	2.614	0.008949
Neutrophils	-2.68E+ 01	2.27E-12	1.82E+ 01	−1.472	0.1411
Mast.cells.activated	−7.38E+ 00	6.21E-04	6.09E+ 00	−1.212	0.225384

### Independent predictive power of eight immune cell types

**Fig. 5:**
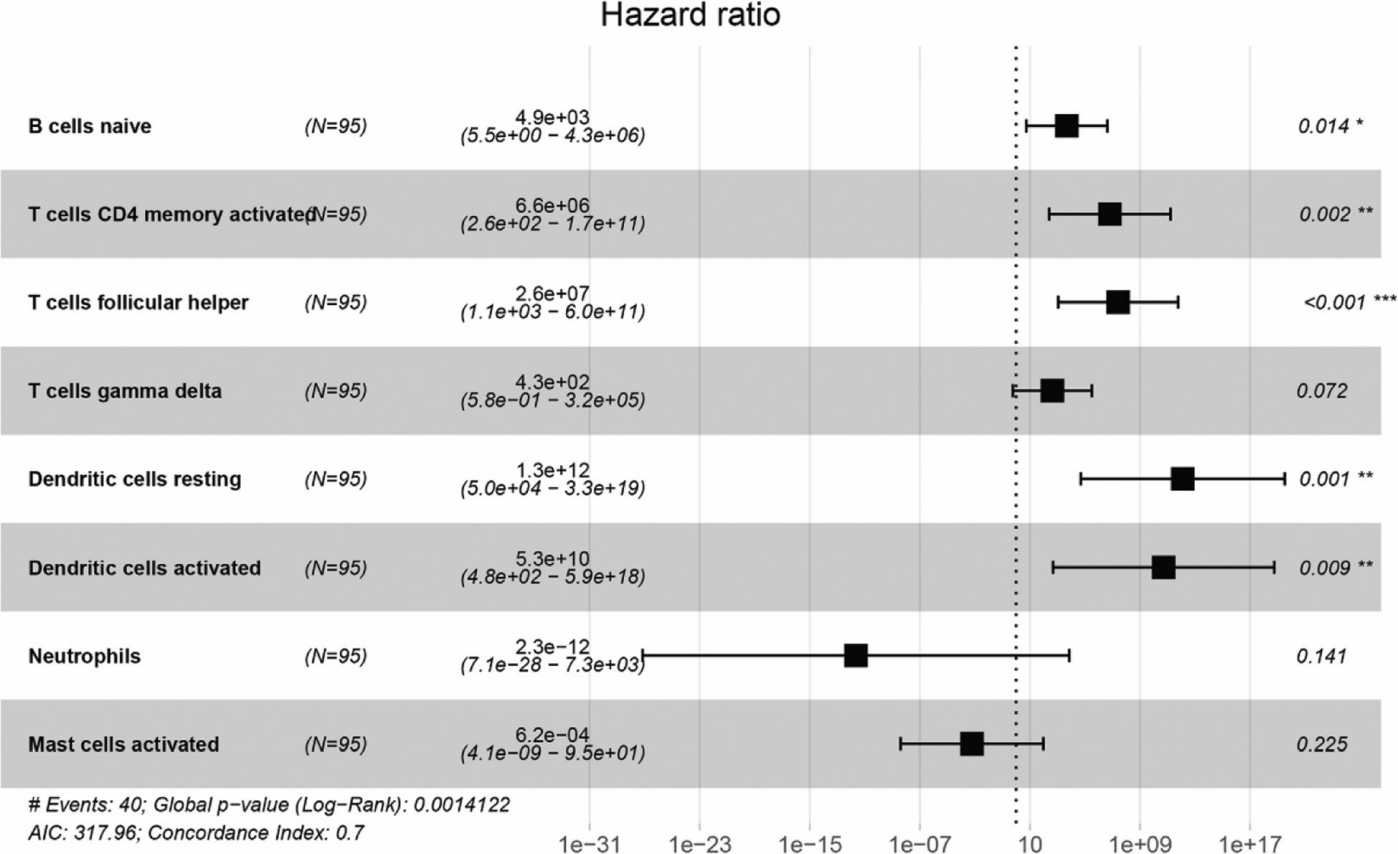
Independent predictive power of eight immune cell types based on multivariate analysis

We also used multivariate analysis to explore whether these 8 immune cell types were independent prognostic factors for osteosarcoma patients. The results indicated that naive B cells, activated memory CD4 + T cells, follicular helper T cells, resting dendritic cells, activated dendritic cells were independent predictors for osteosarcoma patients (Fig. 5).

### Construction of nomogram model

**Fig. 6:**
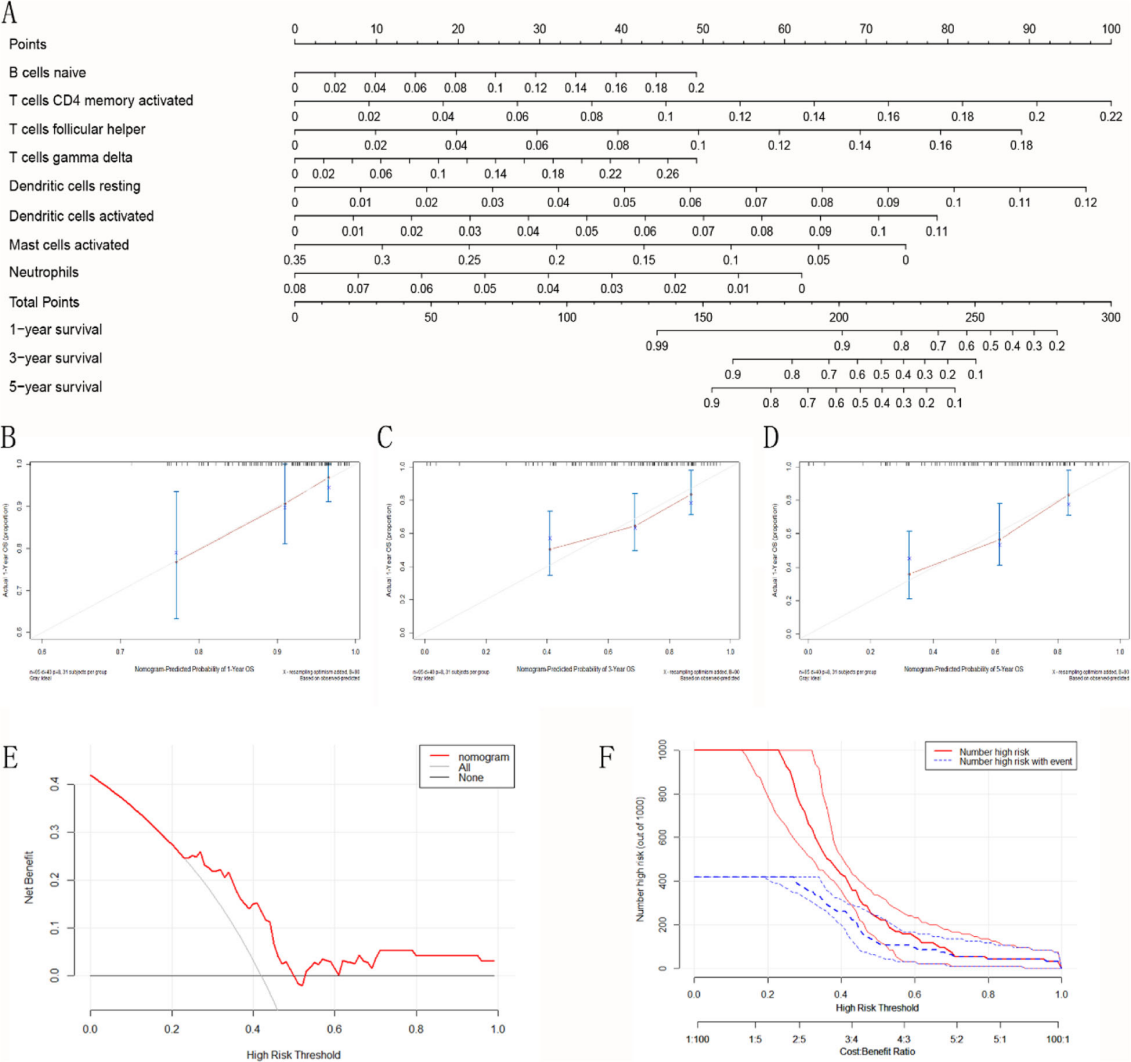
Construction of nomogram model. **a** Nomogram to predict the prognosis of osteosarcoma at 1-, 3- and 5-year based on the eight immune cell types. **b, c, d** Calibration curves to assess the accuracy of the nomogram at 1-, 3- and 5-year. **e** DCA curves assess the clinical value of nomograms. **f** Clinical impact curves evaluate the clinical impact of the nomogram

To assess 1, 3, 5-year survival in patients with osteosarcoma, we constructed a nomogram based on eight screened immune cell types. In the nomogram model, there are several vertical lines passing through the value of each variable, and different integrals can be obtained on the integral line at the top of the nomogram. The integrals of all the variables are added to obtain the total score, and the predicted survival probability values at the corresponding time points are calculated from the line perpendicular to the total score on the prediction line at the bottom of the nomogram. Probability of death = 1- probability of survival. According to the nomogram, we can score the influence of eight immune cell types on the prognosis of patients, and then use the total score to evaluate the 1, 3, 5-year survival rate of patients with osteosarcoma (Fig. 6a). The calibration curve revealed that the predictive ability of the nomogram model at 1-, 3- and 5-year is accurate (Fig. 6b, c, d). The DCA curve revealed that the nomogram model can benefit for the patients (Fig. 6e). Clinical impact curve found that the predictive power of the nomogram model is remarkable (Fig. 6f).

### Biological phenotypes related to immune risk score

**Fig. 7:**
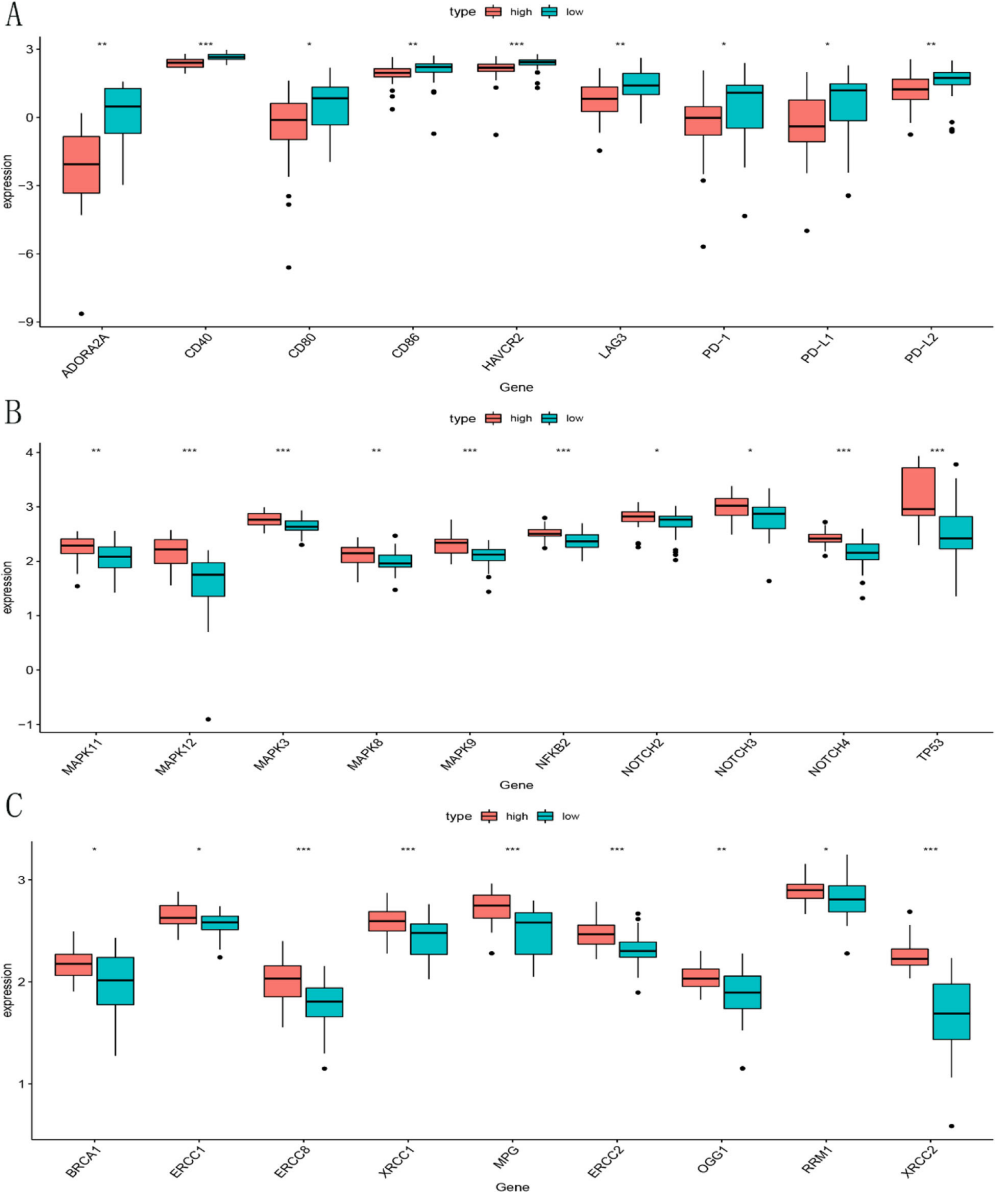
Biological phenotypes related to immune risk score. **a** Comparison of the expression levels of immune checkpoint related genes between high and low risk group. **b** Comparison of the expression levels of cell proliferation related genes between high and low risk group. **c** Comparison of the expression levels of DNA repair related genes between high and low risk group

We explored the expression data in testing dataset and evaluated the correlation between immune risk score and biological phenotypes (immune checkpoint, cell proliferation and DNA repair). We found that the patients in the high risk group had significantly lower expression levels of immune checkpoint related genes than in the low risk group, which indicated that the patients in the low risk group may sensitive to immunotherapy. However, the genes related to cell proliferation and DNA repair were higher expressed in the high risk group (Fig. 7).

## Discussion

Recurrence and metastasis are the main reasons for poor prognosis of osteosarcoma. It is of great significance to accurately predict the recurrence risk of patients and carry out appropriate monitoring and intervention for patients with high recurrence risk. In recent years, increasing researchers began to pay attention to the role of immune microenvironment in tumor development. A recent meta-analysis included more than 120 literature, systematically summarizing the effects of various immune cells such as B cells, NK cells, macrophages and all T cell subsets on the clinical outcomes of tumor patients. The results showed that cytotoxic T cells and memory T cells were beneficial for survival in different tumor types, while the prognosis effect of other immune cells such as B cell, NK cell, macrophages and some helper T cell subsets are associated with tumor type and stage [35]. However, the comprehensive analysis of 22 TIIC types in osteosarcoma is still unsatisfactory. Zhang C et al. [5] used ESTIMATE algorithm to obtain an immune score for each osteosarcoma case from TCGA database, and osteosarcoma cases were divided into the high or low score groups. Difference analysis showed that M0 macrophages and naive B cells were lower in high immune score group than in low immune score group, while M1 macrophages, resting dendritic cells, and M2 macrophages were higher. Then, a risks core model based on different immune related genes was established to predict the prognosis of osteosarcoma. Weifeng Hong et al. [36] used the similar method as Zhang C to investigate the immune infiltration of osteosarcoma from Target database. The result indicated that T cells CD8, T cells CD4 memory activated, M1 macrophages and M2 macrophages were higher in high immune score group than in low immune score group, while Plasma cells, T cells CD4 naive (0.90%), T cells CD4 memory resting, M0 macrophages and Mast cells resting were lower. Deng C et al. [37] used CIBERSORTX algorithm explored the changes of 22 immune cell types infiltration of osteosarcoma from Target database after neoadjuvant chemotherapy. The result indicated that CD8+ T cells, CD3+ T cells, PD-L1+ immune cells and Ki67 + CD8+ T cells in osteosarcoma increased after neoadjuvant chemotherapy. In our research, CIBERSORTX algorithm was used to analyze gene expression in osteosarcoma and obtain the proportion of 22 TIIC types. The results revealed that macrophages account for a large proportion of 22 immune cell types, which were consistent with Morrison C’s study [38]. Then Kaplan-Meier curves were drawn to investigate the prognostic value of 22 TIIC types. The results indicate that naive B cells and Monocytes are associated with poor prognosis of osteosarcoma. As an important component of humoral immunity, B lymphocytes can exert a two-way role in tumor. B lymphocytes can positively regulate tumor immune process by producing anti-tumor antibodies, secreting various cytokines, and acting as antigen-presenting cells, and negatively regulate tumor immune process by inhibiting the proliferation of immune-activated T cells [39]. In addition, studies have shown that elimination of B lymphocytes not only helps to inhibit tumor progression and recurrence, but also significantly increases the sensitivity of patients to chemotherapy [40]. As the precursor of macrophages, monocytes can be recruited into tumor tissues and polarized into M1 macrophages or M2 macrophages in different tumor environments [41]. Sottnik JL [42] and Tuohy JL [43] all revealed that monocytes are negatively associated with disease free survival of canine osteosarcoma. Cersosimo F. et al. reviewed that M2 macrophages was associated with OS metastasis and poor patient prognosis [44], which partly support our analysis results.

At present, several immune risk score models used to quantify the immune status and indicate the prognosis of patients have been proposed in colorectal cancer and breast cancer respectively. These immune risk score models was proposed based on the counts of two lymphocyte populations of tumor centers and invasion margins [45, 46]. The immune risk score model can be used to identify patients with recurrent risk or benefit from corresponding adjuvant chemotherapy. However, there have no studies involve the construction of immune risk score model in osteosarcoma. In our study, we constructed an immune risk score model based on eight immune cell types selected by forward stepwise approach from 22 TIIC types. Different from the traditional immunohistochemical method, the immune cells we used to construct the immune risk score model was screened by CIBERSORTX algorithm. It has more advantages than traditional methods, such as simple operation, accurate results and more immune cell markers. As expecting, our immune risk score model showed a well prognostic value. The patients in the high risk group had a poor prognosis may relate to stronger cell proliferation and repair ability. In conclusion, the immune risk score model has a good clinical application value and worth popularizing. In addition, considering the intuition of the immune risk score model in clinical application, we constructed a nomogram model based on the eight selected immune cell types to present the prediction results simply and clearly. Nomogram is a model that quantifies the probability of an event individually and accurately by integrating multiple predictors based on multi-factor analysis. So far, there is no perfect nomogram model, and the nomogram model in this study can only serve as a reference for clinicians.

Some limitations of this research should be discussed. Firstly, without complete clinical information, in addition to survival and follow-up data, other important clinical information such as age, gender, staging, metastasis and chemotherapy are not available, so that we cannot explore the independence of the prognostic value of the immune risk score model. Besides, it is also impossible to compare the immune risk score model with traditional GTM stage. Secondly, the heterogeneity of the location of immune infiltration was not taken into account in this research. Thirdly, samples were downloaded from different datasets to enlarge the sample size, which may influent the repeatability of outcome. Finally, limited prognostic osteosarcoma data can be searched in the GEO database, so there may be some bias in the results.

## Conclusions

In summary, our study revealed an association between clinical outcomes and immune infiltration patterns in osteosarcoma. The immune risk score model based on eight immune cell types is reliable in predicting the prognosis of osteosarcoma.

## Data Availability

All data generated or analysed during this study are included in this published article and its supplementary information files. GSE16091, GSE21257 and GSE39055 dataset was downloaded from GEO database (https://www.ncbi.nlm.nih.gov/geo/). The testing dataset Target-OS was obtained from the Target database (https://ocg.cancer.gov/programs/target). The public access to the databases is open.

## Abbreviations

TME: Tumor microenvironment
Cox PHR: Cox proportional hazard regression model
ROC: Receiver operating characteristic curve
TIICs: Tumor infiltrating immune cells
GEO: Gene expression omnibus
